# *Fusarium* and *Alternaria* Toxins in Italian Heritage Common Wheat: Influence of Varieties and Alkylresorcinol Content

**DOI:** 10.3390/foods15060970

**Published:** 2026-03-10

**Authors:** Terenzio Bertuzzi, Sabrina Locatelli, Chiara Lanzanova, Helga Cassol, Federico Siboni, Roberta Battaglia, Paola Giorni, Patrizia Vaccino

**Affiliations:** 1 Department of Animal, Food and Nutrition Science (DIANA), Faculty of Agriculture, Food and Environmental Science, Università Cattolica del Sacro Cuore, 29121 Piacenza, Italy; 2 CREA-CI, Consiglio per la Ricerca in Agricoltura e l’Analisi dell’Economia Agraria, Centro di Ricerca Cerealicoltura e Colture Industriali, Via Stezzano 24, 24126 Bergamo, Italy; sabrina.locatelli@crea.gov.it (S.L.); chiara.lanzanova@crea.gov.it (C.L.);; 3 Department of Sustainable Crop Production (DIPROVES), Faculty of Agriculture, Food and Environmental Science, Università Cattolica del Sacro Cuore, 29121 Piacenza, Italy; 4 CREA-CI, Consiglio per la Ricerca in Agricoltura e l’Analisi dell’Economia Agraria, Centro di Ricerca Cerealicoltura e Colture Industriali, s.s. 11 to Torino, km 2.5, 13100 Vercelli, Italy

**Keywords:** deoxynivalenol, *Alternaria* toxins, *Fusarium* toxins, heritage wheat

## Abstract

Forty heritage common wheat varieties were cultivated in four experimental fields over two consecutive years, to evaluate their susceptibility to *Fusarium* and *Alternaria* fungi and the associated mycotoxins. Marked differences in meteorological conditions between the two years (2023 and 2024) significantly influenced mycotoxin occurrence and impact. Overall, heritage varieties exhibited mycotoxin contamination comparable to those reported for modern wheat cultivars grown in nearby areas; interestingly, an opposite trend was observed among trichothecenes and *Alternaria* toxins. Comparing the varieties with each other, very different contamination levels for both mycotoxin groups were observed; some varieties were consistently susceptible across both years, others only in 2024, likely due to frequent precipitations. However, four varieties consistently displayed low levels of both deoxynivalenol and *Alternaria* toxins. Weak correlations among DON and alkylresorcinol ratios were found, showing that, considering only these heritage wheat varieties, alkylresorcinol content could not always predict the susceptibility to mycotoxin contamination.

## 1. Introduction

In the cereal sector, the cultivation of heritage wheat dating back to the first half of the last century is spreading. Even though scientific studies do not support this claim, consumers commonly view these varieties as suitable for individuals with gluten sensitivity, owing to the widespread but incorrect perception that they contain less gluten [1].

This growing trend has led farmers to reintroduce the cultivation of abandoned varieties to foster supply chains with strong territorial value consisting of organic farming and artisanal processing of flours and doughs.

As reported by several authors, cereals can be susceptible to several fungal diseases that can develop during the growing season, often resulting in significant economic losses [2,3]. Moreover, an attack by mycotoxigenic fungi, such as *Fusarium* and *Alternaria* spp., can contaminate the kernels with the related mycotoxins. In northern Italy, the occurrence of deoxynivalenol (DON), the main *Fusarium* toxins in wheat, is often widespread primarily influenced by meteorological conditions, but also by agronomic practices and varietal susceptibility [4,5]. As regards *Alternaria* toxins (ALTs), some studies reported the occurrence of tenuazonic acid (TeA), alternariol (AOH), alternariol mono ether (AME) and tentoxin (TEN) [6,7]. The European Communities fixed different limits for DON in cereals; for common wheat, the limit was recently reduced to 1000 µg/kg in Regulation 2024/1022 [8]. On the other hand, no limits were set for alternariols; to date, Recommendation 2022/553 has indicated benchmark levels for cereal based foods for infants and young children [9]. To reduce disease contamination, several strategies can be adopted in conventional systems, including the use of resistant varieties, fungicides, biological control agents and agronomic/management measures; no fungicides and only some biological agents are applicable under organic farming [10]. Generally, the cultivation of naturally resistant varieties is effective and least expensive [11,12], as well as aligning with the European Parliament and Council Directive 2009/128/EC to achieve the sustainable use of pesticides [13]. Some cereals show a natural resistance to fungal diseases, due to their defense system. As regards the attack of *Fusarium* spp., one of the most common fungal diseases, it has been reported that the alkylresorcinols, naturally present in the bran fraction, are able to defend the plant against the fungal infection and to counteract the presence of related mycotoxins, in particular DON [14]. To our knowledge, no scientific studies were published regarding their efficacy in reducing *Alternaria* infection; similar protective effects could also occur against these mycotoxigenic fungi and their associated toxins.

The aim of this study was to evaluate the apparent resistance of different common wheat heritage varieties to *Fusarium* and *Alternaria* contamination and to assess the occurrence of their related mycotoxins. For this purpose, 40 heritage wheats were cultivated over two consecutive years in four experimental fields located in Northern Italy. After the harvest, kernels were analyzed for fungal incidence, mycotoxins and alkylresorcinol content.

## 2. Materials and Methods

### 2.1. Field Experiments

A total of 40 heritage common wheat varieties (Supplementary Table S1) from the genebank of CREA in Vercelli were cultivated in four experimental fields during the 2022–2023 and 2023–2024 growing seasons. Fields were located in the province of Bergamo (BG), Vercelli (VC), and in 2 sites in the province of Pavia (PV1 and PV2). All fields were managed under organic farming practices. Data on daily temperature and precipitation were measured by meteorological stations located near the experimental fields. The fields were plowed each year, incorporating debris into the soil, and this practice was followed by disk harrowing to prepare a suitable seedbed. Planting was conducted during 23–27 November 2022 and 18–23 November 2023. Each variety was cultivated in three replicated plots (6 m^2^ each). Harvests were carried out manually between 12 and 14 July in both years. Morphophysiological aspects, such as plant height, heading date, lodging and presence of fungal infections (septoria, powdery mildew, and rust) were recorded. After harvest, the samples were threshed and about 50 kernels were randomly selected for fungal incidence determination. The remaining fraction was subsequently ground to wholemeal using a Cyclotec experimental mill (Foss Tecator AB, Höganäs, Sweden) equipped with a 1 mm sieve and kept at −20 °C until the mycotoxin analysis. A subsample was taken from each plot to determine the grain moisture. The grain yield results were adjusted to 13% moisture content.

### 2.2. Fungal Isolation and Mycotoxin Contamination in Harvested Grains

#### 2.2.1. Fungal Incidence

Fifty kernels were disinfected with 1% sodium hypochlorite solution (NaClO) for 2 min followed by 80% ethyl alcohol solution for another 2 min and then rinsed with sterile distilled water for 5 min. When dried, kernels were transferred to Petri dishes containing Potato Dextrose Agar (PDA) and incubated at 25 °C (12 h light/12 h dark) for 7 days. At the end of incubation, the incidence of infected kernels was calculated by evaluating the developed fungal colonies counted out of 50 (the total number of kernels). The same procedure was followed to determine the incidence of the main mycotoxigenic genera; the identification of *Fusarium* spp. and *Alternaria* spp. isolates at genus level was based on observations with binocular microscope (×40). The analysis was conducted in triplicate for each thesis.

#### 2.2.2. Trichothecene Determination

Analyses and standard preparations were performed according to the methods reported by Bertuzzi et al. [5] slightly modified. Briefly, trichothecenes (TCTs), in particular DON, NIV and 15-Ac-DON, were extracted from 25 g of the sample using 100 mL acetonitrile:water 80 + 20 *v*/*v* for 60 min using a rotary shaker; the extract was filtered and purified through a SPE column (Puritox, R-Biopharm Rhône LTD, Glasgow, UK). After evaporation of 1 mL of the purified extract under gentle nitrogen flow and reconstitution in the LC mobile phase (1 mL), the mycotoxins were determined by LC-MS/MS (Vanquish pump and autosampler coupled with TSQ Fortis mass spectrometer, Thermo-Fisher, San Jose, CA, USA). Chromatographic separation was carried out using a Betasil RP-18 column (5 µm particle size, 150 × 2.1 mm, Thermo-Fisher) with a mobile-phase gradient methanol-ammonium acetate 10 mM (pH 6.8) from 90:10 (isocratic 2 min) to 65:35 in 4 min then isocratic for 3 min; gradient to 90:10 in 1 min and isocratic for 6 min (conditioning step). For TCTs, the ionization was carried out with an H-ESI interface (Thermo-Fisher) in negative mode as follows: spray capillary voltage 3.1 kV, sheath and auxiliary gas 35 and 15 psi, respectively, vaporizer temperature 200 °C, temperature of ion transfer tube 270 °C. The fragmentation ions were: 247, 265 and 295 *m*/*z* for DON (parent ion 355 *m*/*z*, adduct with acetate), 277 and 337 *m*/*z* for 15-Ac-DON (parent ion 397 *m*/*z*), 281 and 311 *m*/*z* for NIV (parent ion 371 *m*/*z*); collision gas (Argon) 1.5 psi, collision energy among 12 and 16 V. The limit of detection (LOD) and the limit of quantification (LOQ) were 5 µg/kg and 10 µg/kg, respectively.

#### 2.2.3. Alternaria Toxins Determination

*Alternaria* toxins (ALTs: tenuazonic acid (TeA), alternariol (AOH), alternariol mono-methyl-ether (AME) tentoxin (TEN)) were extracted using acetonitrile:water 80 + 20 *v*/*v* for 60 min using a rotary shaker; after filtration, an aliquot of the extract (0.2 mL) was diluted with 0.6 mL of deionized water and injected in an HPLC-MS/MS system (Thermo-Fisher). ALTs were chromatographed on a HSS-T3 RP-18 column (5 µm particle size, 150 × 2.1 mm; Waters Corp., Milford, MA, USA) using a mobile-phase gradient acetonitrile-water (both acidified with 0.2% formic acid) from 30:70 to 70:30 in 5 min, then isocratic for 4 min; gradient to 30:70 in 1 min and isocratic for 6 min (conditioning step). The ionization was carried out with an ESI interface (Thermo-Fisher) in positive mode as follows: spray capillary voltage 4.5 kV, sheath and auxiliary gas 35 and 12 psi, respectively, vaporizer temperature 200 °C, temperature of ion transfer tube 270 °C. The fragmentation ions were: 125, 139 and 153 *m*/*z* for TeA (parent ion 198 *m*/*z*); 128, 185 and 213 *m*/*z* for AOH (parent ion 259 *m*/*z*); 128, 184 and 258 *m*/*z* for AME (parent ion 273 *m*/*z*); 132, 135 and 312 *m*/*z* for TEN (parent ion 415 *m*/*z*); collision gas (Argon) 1.5 psi, collision energy among 16 and 38 V. The LOD and the LOQ were 0.5 µg/kg and 1.5 µg/kg, respectively, for AOH, AME and TEN, and 2 and 5 µg/kg for TeA.

#### 2.2.4. Alkylresorcinols Determination

Analysis was performed according to the methods reported by Righetti et al. [14]. Briefly, after extraction from milled sample (2.5 g) with 50 mL ethyl acetate for 2 h and filtration, 1 mL was transferred to a screw vial for derivatization step. After evaporation to dryness under a gentle flow of nitrogen and derivatization with 200 μL of hexamethyldisilazane:trimethylclorosilane (80:20, *v*/*v*) for 45 min at 60 °C in subdued light, the extract was again evaporated and redissolved in 1 mL of ethyl acetate and 50 μL of solution of methyl behenate (50 mg L^−1^ in ethyl acetate, used as internal standard). GC-MS analysis was carried out using a Trace GQ Ultra coupled with a Trace GC Ultra single quadrupole mass spectrometry (Thermo-Fisher Scientific, San Jose, CA, USA) using a capillary column Rtx-5MS, 30 m × 0.25 mm i.d., 0.25 μm film thickness (Restek Corporation, Bellefonte, PA, USA). The sample was injected (2 μL, split ratio 1:30) into the GC-MS by a programmed temperature vaporization (PTV) injector in splitless mode. The oven temperature programming was from 120 °C (held for 1 min) to 320 °C at 15 °C/min (held for 7 min). Fragment ions monitored were *m*/*z* 147, 207, 268, 281, 492, 520, 548, 576 for ARs; 311 and 354 for internal standard.

#### 2.2.5. Statistical Analysis of Data

Incidence of fungal presence was arcsine-transformed, while mycotoxin contamination was ln-transformed before statistical analysis [15,16]. All data obtained were subjected to univariate analysis of variance (ANOVA) using the generalized linear model (GLM) procedure, and significant differences between means were confirmed using Tukey’s test. The statistical package IBM SPSS statistics 29 (IBM Corp., Armonk, NY, USA) was used for data analysis.

## 3. Results

### 3.1. Morphophysiological Results

Across all fields and in both the growing seasons, the average plant height was 121 cm, with the Cantore variety showing the highest mean value (143 cm) and Brescia the lowest (85 cm). As expected, taller varieties exhibited higher lodging rates, reaching up to 80%. Grain moisture and yield, adjusted to 13% moisture content, varied considerably: moisture ranged from 11.9% to 15.2%, while grain yield ranged from 0.78 t/ha to 4.86 t/ha. In Supplementary Table S2, average data for each variety are shown.

### 3.2. Fungal Infection

From a statistical point of view, significant differences were found in fungal incidence among the different wheat varieties only considering Fusaria species (*p* ≤ 0.05), while no differences were found for Alternaria species and for the total fungal presence (*p* ≥ 0.05); however, significant differences among heritage varieties were also found for mycotoxins contamination, both DON (*p* ≤ 0.05) and TeA (*p* ≤ 0.01) (Table 1).

Although the differences among varieties and between years were not significant for total fungal contamination from a statistical point of view, it was possible to observe different levels of fungal contamination; in particular, the varieties Attilio Fabrini and Damiano Chiesa showed the lowest contamination (<60%) in 2023, while Ardito resulted the least contaminated in 2024. The highest presence of fungi (100% of seeds showing fungal development) was registered in 2024 by the varieties Damiano Cremona, Dante, Fiume, Gentil rosso 13 and Mentana, while only by the variety Ardito (Supplementary Figure S1) in 2023. Among locations, the highest fungal incidence was observed in VC (98.3%), the lowest in BG (72.1%).

**Figure 1:**
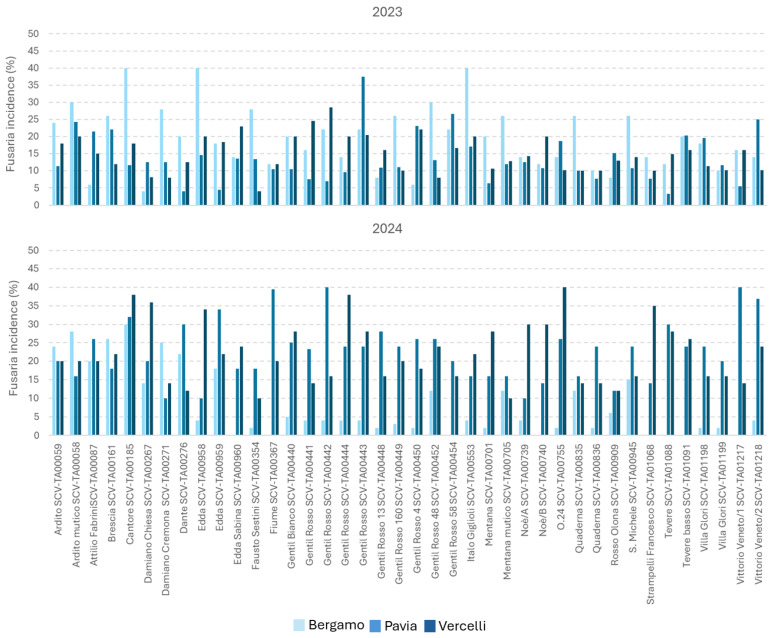
*Fusaria* incidence in the 40 heritage wheat varieties analyzed in this study during years 2023 and 2024. For Pavia, the two fields considered were represented as mean in the graph. Incidence was calculated as the number of fungal isolates belonging to the genus *Fusarium* divided by the total number of kernels analyzed.

**Figure 2:**
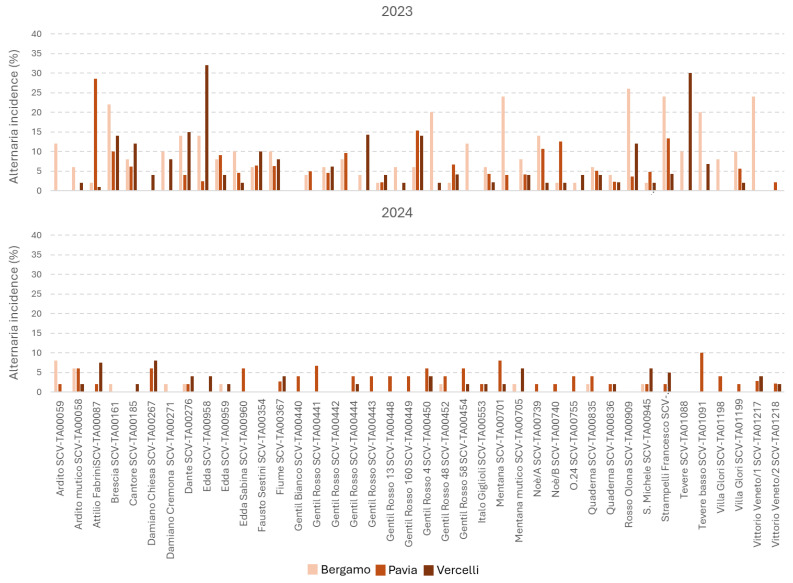
*Alternaria* spp. incidence in the 40 heritage wheat varieties analyzed in this study during years 2023 and 2024. For Pavia, the two fields considered were represented as mean in the graph. Incidence was calculated as the number of fungal isolates belonging to the genus *Alternaria* divided by the total number of kernels analyzed.

Considering only *Fusarium* spp. and *Alternaria* spp. incidence, the contamination of these mycotoxigenic genera was significantly different in the two years considered, but in an opposite way; contamination by *Fusarium* was higher in 2024, while contamination by *Alternaria* was higher in 2023 (Table 1). The Edda varieties (TA00958 and TA00959) showed the lowest *Fusarium* contamination (10%), while Cantore was the most susceptible (28%). As for *Alternaria* spp., the incidence was certainly lower, with infections among the varieties ranging from 1 to 10%. The location resulted in playing a role in *Fusaria* contamination with Bergamo resulting the less contaminated by these genera, while no differences were underlined for *Alternaria* species. The variability in *Fusarium* and *Alternaria* spp. over the two years is shown in Figure 1 and Figure 2, respectively.

### 3.3. TCTs Occurrence

In both years, only DON was detected, while NIV and 15-Ac-DON were always under LOD. Significant differences were found among heritage varieties (*p* ≤ 0.05) with Ardito being the least contaminated and Fausto Sestini the most contaminated. Among locations, Pavia was the least contaminated, while 2024 was the most conducive year for DON production (Table 1). In 2023, the contamination with DON was widespread but at low levels; the values for each field are reported in Supplementary Table S2. Similar values were found in BG and VC fields, while very low values, often below the LOD, were detected in PV1 and PV2 fields. In 2024, spring was characterized by heavy rainfall, causing an increase in DON contamination for all the varieties considered (Supplementary Table S2). In the BG field, three varieties exceeded the legal limit of 1000 µg/kg for unprocessed common wheat intended for human consumption; on the contrary, ten varieties showed levels below 200 µg/kg. As regards the VC field, four varieties exceeded the legal limit, while twelve varieties showed levels lower than 200 µg/kg; in seven of these, the levels were below the LOQ (10 µg/kg). The DON contamination in PV fields was much lower compared to BG and VC fields, although increasing respectively in 2023. Table 2 shows the average values of the four fields for each variety; considering both years, the varieties always showing DON levels lower than 100 µg/kg were Ardito, Dante, O.24, Quaderna (TA00835), Tevere and Villa Glori (TA01198).

### 3.4. Alternaria Toxins Occurrence

In both years, TeA was the most widespread *Alternaria* toxin (Supplementary Table S3); TEN was always detected at low levels, while AOH and AME often showed levels below LOD. Statistical analysis was conducted only on TeA contamination, with results significantly influenced by the heritage variety (*p* ≤ 0.01) and by the location (*p* ≤ 0.01) but not by the year (Table 1). The most contaminated variety was Mentana, while the lowest was Damiano Cremona. The most contaminated fields were BG and VC fields. Unlike DON, TeA contamination was slightly higher in 2023 than 2024, even if differences were not statistically relevant. Considering AOH levels, it is possible to observe a widespread contamination in the BG field for both years and in the PV2 field in 2024. There are currently no EU legal limits for cereals not directly intended for human consumption; for the cereal supply chain, EU Recommendation 2022/553 indicated values only for cereal based foods for infants and young children [9]. Six varieties (Ardito, Damiano Cremona, Dante, Gentil Rosso 48, Quaderna—TA00835 and Villa Glori—TA01198,) showed average TeA contamination below 50 µg/kg in both years and in all fields (Table 2); among these varieties, Ardito, Damiano Cremona and Quaderna TA00835 also showed low levels for TEN (<5 µg/kg), AOH (<2 µg/kg) and AME (<LOD).

### 3.5. Alkylresorcinol and Mycotoxin Content

The alkylresorcinol (AR) levels found in this study were very different among the varieties; moreover, the same variety often showed different levels in the different experimental fields; then, no significant difference was found among the varieties due to high variability. However, the ratios among the AR concentrations for each variety, even if cultivated in the different experimental fields, were fairly constant. Previous studies investigated the correlation between ARs ratio and *Fusarium* incidence or DON contamination. Ciccoritti et al. [17] reported the ratio of AR21/23 as an antifungal activity indicator on FHB incidence in vitro experiments, and Ziegler et al. [18] evaluated the AR 21/17 as a genetics-related indicator in different *Triticum* spp., having different degrees of ploidy. Finally, Righetti et al. [14] found that, comparing wheat cultivars belonging to different genotypes and ploidy levels, DON contamination was negatively correlated with AR21/23, while DON-3-Glc was correlated with the ratio of AR17:0/AR21:0. Taking into consideration the results of these studies, it was investigated whether the AR ratios in different varieties of common wheat could be correlated to DON or *Alternaria* toxins contamination. For this purpose, only the fields of Vercelli and Bergamo were considered, since in the other fields, the mycotoxin contamination was very low and often not detected. No significant correlation was found for each AR ratio; only weak correlations were found (from 0.2746 to 0.3685; *p* < 0.05) for AR21/17 and AR21/19 ratios in some fields in both 2023 and 2024. Similarly, for *Alternaria* toxins, weak or no correlations were found between the TeA + TEN and AR ratios (maximum value r = 0.3912 for AR21/19; *p* < 0.05).

## 4. Discussion

The two years under study exhibited markedly different spring weather conditions, a critical period for mycotoxigenic fungal contamination in wheat. In 2023, rainfall was moderate and temperatures were relatively high for the season, whereas in 2024, precipitation was abundant and often intense, accompanied by temperatures below the seasonal average. This led to different contamination levels in the two years, especially regarding fungal presence and mycotoxin occurrence. Most heritage wheat varieties showed satisfactory resistance to DON and ALT production in both years although this did not always correlate with similar resistance to fungal contamination for the same varieties. The EU legal limit for DON was exceeded only in a few varieties. The DON contamination levels found in these heritage varieties were compared with those of modern wheat cultivated under conventional system in adjacent fields and nearby areas, showing values ranging from <LOD to 316.4 µg/kg and from 102.4 to 678.9 µg/kg in 2023 and 2024, respectively; as reported by [19], the DON contamination in 2023 was not concerning in wheat produced in northern Italy, while it was high in 2024 in several modern wheat lots. *Alternaria* toxins are considered a group of emerging mycotoxins, not yet regulated in EU and in several countries; only benchmark levels for TeA, AOH and AME in cereal based foods for infants and young children were reported in EU Recommendation 2022/553 [9]. Several studies investigated the occurrence of *Alternaria* toxins in wheat, reporting TeA as the most prevalent compound [20,21,22,23,24,25]; our data agreed with these findings, showing a widespread contamination with TeA but at low levels. To our knowledge, very few scientific papers have investigated mycotoxin contamination in heritage wheat varieties. One of the most relevant and recent contributions is the recent study by Sardella et al. [26], which examined hulled wheats (einkorn, emmer, spelt) and old Italian bread wheat genotypes under multiple environments. Their findings indicated that ancient and old bread wheat genotypes generally showed a reduced susceptibility to mycotoxin accumulation when compared to modern bread wheat varieties. However, the authors also emphasized that the variability was high, with some hulled wheats and old genotypes exhibiting either higher or lower mycotoxin levels depending on environmental conditions and growing sites. Moreover, the study of Tucker et al. [27] reported the use of heritage barley varieties improved the resistance against *Fusarium graminearum* infestation and DON production.

Regarding the trend among the two groups of mycotoxins considered in this study, no correlation was found between DON and *Alternaria* toxins; comparing the two years of the study, an opposite trend was observed between the two mycotoxin groups. This behavior was probably influenced by meteorological conditions: in 2023, non-persistent precipitations occurred in spring limiting DON production but favoring alternariol occurrence; on the contrary, frequent heavy rains in 2024 spring favored DON contamination. Moreover, competition among the two fungal genera should be considered in future studies. Jevtić et al. [28] reported that an infection level of *F. graminearum* over 25% showed antagonistic activity against *Alternaria* spp. under field conditions; Müller et al. [29] found that isolates of either *Alternaria* or *Fusarium* strains showed weak growth if inoculated together. When comparing the heritage varieties, very different contamination levels for both TCTs and ALTs were observed; some varieties showed susceptibility to mycotoxin contamination in both years, while others did only in 2024 due to frequent precipitations. Notably, four varieties—Dante, Quaderna TA00835, Villa Glori and Ardito—were always less contaminated by both DON and ALTs. All four varieties were developed by Nazareno Strampelli in the early 20th century and are phylogenetically similar, almost sharing the same pedigree, which originates from the cross Wilhelmina Tarwe/Rieti/Akagomugi. On the contrary, most Gentil Rosso accessions showed not negligible levels of mycotoxins; only Gentil Rosso 48 showed low levels in both years. These accessions, characterized by tall stature and resistance to rust disease, originated in the Tuscany region and were cultivated in northern Italy until the First World War. Generally, the varieties most resistant to TCTs and ALTs were characterized by a plant height below 120 cm, low lodging, grain yield higher than 3.5 t/ha and grain moisture content at harvest below 13%. The weak correlations observed between DON and alkylresorcinol ratios showed that AR ratios are probably not a reliable index to predict the susceptibility of different common wheat varieties to mycotoxin contamination.

## 5. Conclusions

The reintroduction into cultivation of heritage wheat varieties requires an assessment of their susceptibility to mycotoxin contamination. This study showed different levels of resistance among the evaluated varieties, enabling the identification of those most suitable for cultivation under organic farming systems. Further detailed investigations should be carried out to elucidate the causes of different resistance obtained by the varieties, given that all were cultivated using the same agronomic practices and subjected to the same weather conditions.

## Figures and Tables

**Table 1:** Analysis of variance (ANOVA) of main fungal genera presence (*Fusaria* incidence (%) and *Alternaria* incidence (%)) and mycotoxin contamination (deoxynivalenol and tenuazonic acid) in the different heritage varieties and locations and in the two years considered in the study (2023 and 2024). Different letters mean significant differences according to Tukey’s test. The 2 fields considered in Pavia were managed as mean value for the statistical analysis.

	Total Fungi Incidence (%)	*Fusaria* Incidence (%)	*Alternaria* Incidence (%)	DON (µg/kg)	TeA (µg/kg)
**Variety (A)**	**n.s.**	*	**n.s.**	*	**
Ardito	76.0	23.0 ^AB^	3.7	18.9 ^C^	31.7 ^CDE^
Ardito mutico	80.0	19.6 ^AB^	3.7	264.7 ^ABC^	246.9 ^AB^
Attilio Fabrini	70.1	18.1 ^AB^	6.8	224.5 ^ABC^	47.1 ^BCDE^
Brescia	96.3	21.0 ^AB^	8.0	474.6 ^AB^	43.7 ^BCDE^
Cantore	71.5	28.3 ^A^	4.7	320.2 ^AB^	55.9 ^BCDE^
Damiano Chiesa	69.7	15.8 ^AB^	3.0	193.0 ^ABC^	36.8 ^CDE^
Damiano Cremona	82.7	16.3 ^AB^	3.3	128.6 ^ABC^	17.5 ^E^
Dante	91.0	16.8 ^AB^	6.8	74.9 ^ABC^	31.6 ^DE^
Edda	89.1	11.2 ^B^	2.3	152.2 ^ABC^	157.3 ^BCD^
Edda Sabina	91.7	17.6 ^AB^	8.6	145.3 ^ABC^	99.3 ^BCD^
Fausto Sestini	91.7	20.4 ^AB^	8.7	549.4 ^A^	123.3 ^ABCD^
Fiume	83.0	19.2 ^AB^	4.2	359.5 ^ABC^	52.0 ^BCDE^
Gentil Bianco	72.3	15.4 ^AB^	3.8	316.7 ^AB^	68.6 ^BCDE^
Gentil Rosso	85.1	15.3 ^AB^	3.0	450.7 ^AB^	126.1 ^ABCD^
Gentil Rosso 13	86.8	19.6 ^AB^	2.8	116.7 ^ABC^	69.9 ^BCD^
Gentil Rosso 160	86.0	18.3 ^AB^	3.9	144.5 ^ABC^	68.9 ^BCD^
Gentil Rosso 4	77.0	22.7 ^AB^	3.7	271.6 ^ABC^	90.1 ^BCD^
Gentil Rosso 48	87.0	13.5 ^B^	2.0	138.4 ^AB^	44.9 ^BCDE^
Gentil Rosso 58	92.7	15.7 ^AB^	2.0	567.6 ^ABC^	101.8 ^BCD^
Italo Giglioli	85.7	16.2 ^AB^	7.6	123.9 ^ABC^	59.8 ^BCDE^
Mentana	94.7	18.9 ^AB^	4.7	329.4 ^AB^	619.9 ^A^
Mentana mutico	78.0	16.9 ^AB^	3.5	381.1 ^ABC^	132.4 ^BCD^
Noè/A	95.7	19.8 ^AB^	2.7	391.9 ^AB^	81.6 ^BCD^
Noè/B	82.7	13.8 ^B^	3.7	172.0 ^ABC^	87.6 ^BCD^
O.24	89.0	14.8 ^AB^	6.0	97.3 ^ABC^	107.4 ^BCD^
Quaderna	77.2	14.3 ^AB^	3.9	159.5 ^ABC^	51.9 ^BCDE^
Rosso Olona	75.3	18.5 ^AB^	3.4	222.5 ^AB^	64.6 ^BCD^
S. Michele	91.5	14.7 ^AB^	2.0	185.4 ^ABC^	101.2 ^BCD^
Strampelli Francesco	82.0	13.5 ^B^	2.6	373.1 ^ABC^	153.9 ^ABC^
Tevere	90.7	14.7 ^B^	6.9	24.6 ^BC^	98.6 ^BCD^
Tevere basso	75.8	17.7 ^AB^	8.3	137.3 ^ABC^	73.2 ^BCD^
Villa Glori	85.5	13.4 ^B^	3.4	149.8 ^ABC^	49.4 ^BCDE^
Vittorio Veneto/1	75	15.3 ^AB^	4.1	226.1 ^ABC^	60.7 ^BCDE^
Vittorio Veneto/2	74.2	19.0 ^AB^	4.7	117.6 ^ABC^	74.9 ^BCDE^
**Field (B)**	**	**	**n.s.**	**	**
Bergamo	72.1 ^C^	13.5 ^B^	5.2	285.8 ^A^	111.9 ^A^
Pavia (2)	77.6 ^B^	18.2 ^A^	3.9	0.68 ^B^	62.0 ^B^
Vercelli	98.3 ^A^	18.5 ^A^	4.0	399.7 ^A^	123.6 ^A^
**Year (C)**	**n.s.**	*	**	*	**n.s.**
2023	81.0	16.0 ^B^	6.8 ^A^	164.5 ^B^	119.7
2024	85.9	18.1 ^A^	1.8 ^B^	328.5 ^A^	78.7
AxB	*	**	*	**n.s.**	**n.s.**
AxC	**n.s.**	**n.s.**	**n.s.**	**n.s.**	**n.s.**
BxC	**n.s.**	**	**	**	**
AxBxC	**n.s.**	*	**n.s.**	**n.s.**	**n.s.**

n.s.: not significant, *: *p* ≤ 0.05; **: *p* ≤ 0.01.

**Table 2:** DON and *Alternaria* toxins’ average values (µg/kg) for each heritage wheat variety cultivated in four different locations in 2023 and 2024.

		Mean ± SD DON 2023	Mean ± SD DON 2024	Mean ± SD TeA 2023	Mean ± SD TeA 2024	Mean ± SD TEN 2023	Mean ± SD TEN 2024	Mean ± SD AOH 2023	Mean ± SD AOH 2024	Mean ± SD AME 2023	Mean ± SD AME 2024
Ardito	TA00059	10.0 ± 1.1	32.1 ± 54.3	25.0 ± 13.7	41.0 ± 14.2	3.6 ± 0.9	3.8 ± 1.1	0.3 ± 0.1	0.5 ± 0.1	0.3 ± 0.1	0.3 ± 0.1
Ardito mutico	TA00062	59.5 ± 67.6	342.5 ± 485.3	250.8 ± 307.2	207.6 ± 308.0	21.3 ± 4.0	13.4 ± 3.5	1.4 ± 0.5	0.4 ± 0.1	0.5 ± 0.1	0.3 ± 0.1
Attilio Fabrini	TA00087	66.8 ± 74.9	274.9 ± 349.6	49.7 ± 53.2	58.8 ± 58.7	1.6 ± 0.4	9.7 ± 3.3	0.3 ± 0.1	0.4 ± 0.1	0.3 ± 0.1	0.3 ± 0.1
Brescia	TA00161	94.1 ± 97.5	621.5 ± 819.8	50.0 ± 26.4	44.8 ± 12.5	4.5 ± 2.7	14.5 ± 5.7	0.4 ± 0.1	3.8 ± 1.4	0.3 ± 0.1	0.9 ± 0.2
Cantore	TA00185	242.5 ± 411.5	170.4 ± 202.7	73.4 ± 73.2	23.8 ± 9.1	5.8 ± 4.5	6.7 ± 4.1	0.8 ± 0.3	2.1 ± 1.4	0.3 ± 0.1	0.3 ± 0.1
Damiano Chiesa	TA00267	39.0 ± 68.1	256.7 ± 302.2	22.8 ± 24.5	54.0 ± 59.2	3.6 ± 1.6	5.2 ± 4.1	0.3 ± 0.1	0.6 ± 0.2	0.3 ± 0.1	0.3 ± 0.1
Damiano Cr	TA00271	56.3 ± 97.5	141.6 ± 187.7	18.1 ± 11.9	19.0 ± 16.0	3.2 ± 1.8	4.3 ± 1.9	0.6 ± 0.2	1.7 ± 1.2	0.3 ± 0.1	0.3 ± 0.1
Dante	TA00276	20.8 ± 31.5	99.9 ± 66.9	30.7 ± 35.2	26.3 ± 8.7	4.8 ± 4.2	6.0 ± 5.1	1.3 ± 0.5	3.0 ± 1.5	0.3 ± 0.1	0.3 ± 0.1
Edda	TA00958	126.4 ± 149.2	146.8 ± 132.7	89.5 ± 89.1	45.1 ± 14.2	10.1 ± 4.6	10.8 ± 5.1	0.3 ± 0.1	0.7 ± 0.3	0.3 ± 0.1	0.3 ± 0.1
Edda	TA00959	34.1 ± 49.8	135.8 ± 151.0	270.9 ± 402.6	115.2 ± 118.4	11.0 ± 6.2	8.9 ± 6.2	0.6 ± 0.2	2.7 ± 2.1	0.3 ± 0.1	0.3 ± 0.1
Edda Sabina	TA00960	69.0 ± 76.2	155.2 ± 300.4	116.3 ± 123.9	60.5 ± 27.4	10.4 ± 5.8	7.8 ± 4.9	0.3 ± 0.1	0.6 ± 0.2	0.3 ± 0.1	0.3 ± 0.1
Fausto Sestini	TA00354	128.4 ± 130.3	593.0 ± 878.2	100.4 ± 82.9	133.0 ± 42.4	9.2 ± 5.3	17.9 ± 11.4	1.1 ± 0.5	4.4 ± 4.0	0.7 ± 0.3	3.0 ± 2.5
Fiume	TA00367	305.3 ± 557.0	238.9 ± 273.3	54.5 ± 72.0	43.2 ± 35.5	3.9 ± 2.2	6.0 ± 6.2	0.4 ± 0.1	2.7 ± 2.0	0.3 ± 0.1	0.3 ± 0.1
Gentil Bianco	TA00440	196.8 ± 221.4	211.9 ± 247.7	67.0 ± 94.2	50.4 ± 10.3	7.9 ± 7.1	7.6 ± 5.0	0.3 ± 0.1	1.7 ± 0.6	0.3 ± 0.1	1.4 ± 0.7
Gentil Rosso	TA00441	130.3 ± 156.0	327.8 ± 425.5	156.5 ± 163.7	159.1 ± 236.6	5.4 ± 5.0	8.4 ± 5.7	0.7 ± 0.2	2.0 ± 1.8	0.3 ± 0.1	0.3 ± 0.1
Gentil Rosso	TA00442	43.2 ± 64.6	563.8 ± 767.5	85.8 ± 93.6	81.7 ± 12.1	8.1 ± 6.7	15.1 ± 8.4	1.1 ± 0.6	2.1 ± 0.2	2.2 ± 0.4	0.3 ± 0.1
Gentil Rosso	TA00443	475.9 ± 614.1	418.1 ± 674.7	169.9 ± 150.3	81.2 ± 33.9	10.9 ± 4.2	10.6 ± 8.5	0.6 ± 0.2	1.7 ± 0.5	0.3 ± 0.1	0.3 ± 0.1
Gentil Rosso	TA00444	202.6 ± 285.0	371.7 ± 510.0	74.5 ± 62.6	85.3 ± 82.3	9.6 ± 9.2	13.4 ± 12.8	0.3 ± 0.1	0.7 ± 0.2	0.3 ± 0.1	0.3 ± 0.1
Gentil Rosso 13	TA00448	41.4 ± 54.4	138.7 ± 159.4	70.5 ± 29.7	88.4 ± 66.7	6.4 ± 5.1	10.6 ± 8.8	0.3 ± 0.1	2.0 ± 1.2	0.3 ± 0.1	2.9 ± 2.6
Gentil Rosso 160	TA00449	161.0 ± 190.4	62.1 ± 114.2	65.9 ± 32.5	64.4 ± 40.5	11.2 ± 4.3	7.7 ± 6.5	0.3 ± 0.1	1.3 ± 0.4	0.3 ± 0.1	0.3 ± 0.1
Gentil Rosso 4	TA00450	176.7 ± 343.4	223.8 ± 201.6	108.0 ± 94.8	48.8 ± 19.5	7.5 ± 8.1	6.0 ± 4.3	2.4 ± 2.5	1.5 ± 1.3	3.5 ± 1.7	2.6 ± 2.3
Gentil Rosso 48	TA00452	70.9 ± 131.8	142.9 ± 166.1	43.6 ± 37.2	41.1 ± 22.6	8.3 ± 7.9	13.7 ± 12.4	0.3 ± 0.1	0.6 ± 0.2	0.3 ± 0.1	0.3 ± 0.1
Gentil Rosso 58	TA00454	493.7 ± 681.7	362.8 ± 426.7	108.0 ± 118.2	84.5 ± 76.5	6.9 ± 6.5	10.0 ± 8.2	0.3 ± 0.1	2.4 ± 2.0	0.3 ± 0.1	0.3 ± 0.1
Italo Giglioli	TA00553	50.9 ± 72.4	139.9 ± 171.4	70.9 ± 56.3	46.0 ± 22.3	13.1 ± 8.6	10.0 ± 9.8	0.3 ± 0.1	4.0 ± 1.1	0.3 ± 0.1	2.9 ± 1.8
Mentana	TA00701	79.9 ± 81.8	358.8 ± 461.0	569.0 ± 774.2	486.6 ± 89.1	28.5 ± 19.2	19.1 ± 15.8	1.1 ± 1.0	7.5 ± 6.1	0.7 ± 0.2	8.4 ± 5.4
Mentana mutico	TA00705	51.8 ± 62.0	524.8 ± 747.9	156.2 ± 207.2	76.6 ± 57.6	8.7 ± 6.6	5.1 ± 3.1	0.6 ± 0.2	5.6 ± 3.5	0.3 ± 0.1	1.3 ± 0.5
Noè/A	TA00739	177.9 ± 291.8	320.6 ± 393.8	97.4 ± 110.8	48.6 ± 7.2	11.1 ± 9.2	9.1 ± 7.8	0.3 ± 0.1	2.5 ± 2.1	0.3 ± 0.1	1.2 ± 0.9
Noè/B	TA00740	133.6 ± 200.4	129.4 ± 192.2	76.3 ± 102.7	98.7 ± 78.3	5.3 ± 3.0	9.2 ± 7.6	0.3 ± 0.1	1.2 ± 0.6	0.3 ± 0.1	0.3 ± 0.1
O.24	TA00755	70.2 ± 105.4	80.8 ± 90.2	79.5 ± 109.9	129.1 ± 118.4	15.5 ± 12.4	12.4 ± 9.2	0.5 ± 0.1	1.1 ± 1.0	0.3 ± 0.1	0.3 ± 0.1
Quaderna	TA00835	5.0 ± 0.8	67.5 ± 72.5	43.1 ± 9.7	43.6 ± 8.2	3.1 ± 2.4	4.1 ± 1.8	0.4 ± 0.1	0.9 ± 0.4	0.3 ± 0.1	0.3 ± 0.1
Quaderna	TA00836	86.5 ± 130.8	305.3 ± 418.9	52.1 ± 45.1	61.1 ± 13.6	10.5 ± 11.3	10.5 ± 8.9	0.3 ± 0.1	0.9 ± 0.5	0.3 ± 0.1	0.3 ± 0.1
Rosso Olona	TA00909	99.8 ± 113.8	275.4 ± 190.0	68.5 ± 46.9	48.6 ± 14.7	14.4 ± 10.6	13.9 ± 9.7	0.3 ± 0.1	2.0 ± 1.2	0.3 ± 0.1	0.3 ± 0.1
S. Michele	TA00945	146.0 ± 162.8	101.3 ± 168.6	64.6 ± 76.7	116.9 ± 24.1	2.6 ± 2.0	6.9 ± 5.3	0.3 ± 0.1	1.2 ± 0.8	0.3 ± 0.1	1.4 ± 1.1
Strampelli Francesco	TA01068	428.8 ± 758.2	135.9 ± 166.5	187.1 ± 192.7	86.6 ± 34.1	5.9 ± 4.1	4.2 ± 3.9	0.3 ± 0.1	0.3 ± 0.1	0.3 ± 0.1	0.3 ± 0.1
Tevere	TA01088	15.5 ± 21.0	28.9 ± 47.8	79.0 ± 71.2	100.1 ± 73.7	21.9 ± 20.3	17.2 ± 15.2	0.6 ± 0.2	1.6 ± 1.2	0.3 ± 0.1	4.6 ± 4.0
Tevere basso	TA01091	47.0 ± 84.0	142.7 ± 133.4	52.5 ± 42.3	83.8 ± 39.9	5.8 ± 4.2	4.7 ± 4.0	0.3 ± 0.1	1.5 ± 1.3	0.3 ± 0.1	0.3 ± 0.1
Villa Glori	TA01198	20.8 ± 21.5	42.3 ± 74.6	49.8 ± 38.8	37.0 ± 32.7	5.8 ± 2.8	4.0 ± 3.5	0.4 ± 0.1	1.2 ± 0.8	0.3 ± 0.1	0.3 ± 0.1
Villa Glori	TA01199	60.0 ± 87.4	337.5 ± 454.5	57.7 ± 46.5	41.0 ± 14.2	7.2 ± 6.2	4.8 ± 4.1	0.3 ± 0.1	0.7 ± 0.3	0.3 ± 0.1	0.3 ± 0.1
Vittorio Veneto/1	TA01217	150.4 ± 246.8	193.6 ± 260.6	55.9 ± 55.5	51.6 ± 14.6	9.4 ± 6.1	8.7 ± 7.4	0.3 ± 0.1	0.6 ± 0.2	0.3 ± 0.1	0.3 ± 0.1
Vittorio Veneto/2	TA01218	129.1 ± 223.0	53.6 ± 97.2	74.5 ± 72.7	68.0 ± 53.3	7.9 ± 4.8	12.7 ± 9.8	1.2 ± 0.4	0.9 ± 0.4	0.3 ± 0.1	0.3 ± 0.1
Mean		124.8 ± 119.4	248 ± 157.4	99.3 ± 94.5	83.5 ± 76.1	9.0 ± 9.2	9.6 ± 8.8	0.5 ± 0.4	1.8 ± 1.9	0.4 ± 0.1	0.9 ± 0.3

## Data Availability

The original contributions presented in this study are included in the article/Supplementary Materials. Further inquiries can be directed to the corresponding authors.

## Supplementary Materials

The following supporting information can be downloaded at https://www.mdpi.com/article/10.3390/foods15060970/s1. Table S1: List of the heritage common wheat lines analyzed; Table S2: Morphological data for the heritage wheat varieties. For each variety, the values represent the mean across four locations and two growing seasons. Table S3: DON contamination levels (µg/kg) in 2023 and 2024. Table S4: TeA contamination levels (µg/kg) in 2023 and 2024. Figure S1: Total fungal incidence in the 40 heritage wheat varieties analyzed in this study grown in 2023 and 2024. For each variety, the values represent the mean across four locations. Incidence was calculated as the total number of fungal isolates by the total number of kernels analyzed.
