# Supplementary material for: Fusarium and Alternaria Toxins in Italian Heritage Common Wheat: Influence of Varieties and Alkylresorcinol Content
Source: Foods. 2026 Mar 10;15(6):970. doi: 10.3390/foods15060970 (PMC13025222; doi:10.3390/foods15060970)
Supplement: Supplementary file 1 [file foods-15-00970-s001.zip › foods-4129130-supplementary.pdf]

Supplementary Table S1. List of the heritage common wheat lines analysed.

Accession name	Accession number	Ancestry	DOI
Ardito	TA00059	Wilhelmina Tarwe/Rieti ar.21//Akagomughi	10.18730/1KHJJ9
Ardito mutico	TA00062		10.18730/1KHJNC
Attilio Fabrini	TA00087	Wilhelmina Tarwe/Rieti m.67//Akagomughi	10.18730/1KHP08
Brescia	TA00161	Vittorio Niccoli/Precocissimo Cinese	10.18730/1KHYW~
Cantore	TA00185	Triticum villosum/Rieti	10.18730/1KJ28Y
Damiano Chiesa	TA00267	Wilhelmina Tarwe/Rieti m. 67/Akagomughi	10.18730/1KHJWK
Damiano Cremona	TA00271	n.d.	10.18730/1KHK0Q
Dante	TA00276	Wilhelmina Tarwe/Rieti m. 67/Akagomughi	10.18730/1KHK5W
Edda	TA00958	Rieti/Wilhelmina Tarwe	10.18730/1KHY5A
Edda	TA00959	Rieti/Wilhelmina Tarwe	10.18730/1KHZGG
Edda Sabina	TA00960	Rieti/Wilhelmina Tarwe	10.18730/1KHZHH
Fausto Sestini	TA00354	Wilhelmina Tarwe/Rieti ar.21//Akagomughi	10.18730/1KM2A8
Fiume	TA00367	Akagomughi/Carlotta Strampelli	10.18730/1KM41T
Gentil Bianco	TA00440	Landrace	10.18730/1KKH8H
Gentil Rosso	TA00441	Landrace	10.18730/1KKH9J
Gentil Rosso	TA00442	Landrace	10.18730/1KKHAK
Gentil Rosso	TA00443	Landrace	10.18730/1KKHBM
Gentil Rosso	TA00444	Landrace	10.18730/1KKHCN
Gentil Rosso 13	TA00448	Landrace	10.18730/1KKHGS
Gentil Rosso 160	TA00449	Landrace	10.18730/1KJSF~
Gentil Rosso 4	TA00450	Landrace	10.18730/1KJSG$
Gentil Rosso 48	TA00452	Landrace	10.18730/1KJSJU
Gentil Rosso 58	TA00454	Landrace	10.18730/1KJSM1
Italo Giglioli	TA00553	Wilhelmina Tarwe/Rieti m. 67/Akagomughi	10.18730/1KK5TN
Mentana	TA00701	Wilhelmina Tarwe/Rieti ar. 21/Akagomughi	10.18730/1KJVZ2
Mentana mutico	TA00705	Wilhelmina Tarwe/Rieti m. 67/Akagomughi	10.18730/1KJXDB
Noè/A	TA00739	n.d.	10.18730/1KK13J
Noè/B	TA00740	n.d.	10.18730/1KK14K
O. 24	TA00755	n.d.	10.18730/1KK2X2
Quaderna	TA00835	Selection from Mentana	10.18730/1KJKFT
Quaderna	TA00836	Selection from Mentana	10.18730/1KJKGV
Rosso Olona	TA00909	Landrace	10.18730/1KHRPM
S. Michele	TA00945	Wilhelmina Tarwe/Rieti ar. 21/Akagomughi	10.18730/1KHXR$
Strampelli Francesco	TA01068	Hizakiri/Gregorio Mendel	10.18730/1KHG67
Tevere	TA01088	Hatif Inversable/Rieti//Ardito	10.18730/1KHJ4*
Tevere basso	TA01091	Selection from Tevere	10.18730/1KHJ7=
Villa Glori	TA01198	Wilhelmina Tarwe/Rieti m. 67/Akagomughi	10.18730/1KKWKA
Villa Glori	TA01199	Wilhelmina Tarwe/Rieti m. 67/Akagomughi	10.18730/1KKWMB
Vittorio Veneto/ 1	TA01217	Hizakiri/Gregorio Mendel	10.18730/1KKYE*
Vittorio Veneto/ 2	TA01218	Hizakiri/Gregorio Mendel	10.18730/1KKYF~

Supplementary Table S2. Morphological data for the ancient wheat varieties. For each variety, the values represent the mean across four locations and considering both the growing seasons.

		Heading date (days from 01/04)	Lodging	Plant height	Kernel moisture at harvest	Yield
Variety			1-9	cm	%	t/ha
Ardito	TA00059	24	0	110	12,3	3,80
Attilio Fabrini	TA00087	51	6	150	15,2	0,78
Brescia	TA00161	29	1	90	12,4	4,27
Cantore	TA00185	42	6	155	12,8	1,92
Damiano Chiesa	TA00267	28	0	100	12,3	3,68
Damiano Cremona	TA00271	26	0	105	11,9	4,55
Dante	TA00276	33	8	120	12,2	3,52
Edda	TA00958	20	0	105	12,4	2,99
Edda Sabina	TA00960	20	0	105	12,2	3,10
Edda/ 1	TA00959	20	0	110	12,4	2,08
Fausto Sestini	TA00354	33	7	125	12,2	2,06
Fiume	TA00367	42	8	150	12,4	1,87
Gentil Bianco	TA00440	44	5	135	12,5	2,69
Gentil Rosso	TA00441	42	9	160	12,4	2,30
Gentil Rosso	TA00442	42	8	135	12,5	1,98
Gentil Rosso	TA00444	42	9	145	12,8	1,86
Gentil Rosso	TA00443	42	9	130	13,0	1,58
Gentil Rosso 13	TA00448	34	5	130	12,4	2,96
Gentil Rosso 160	TA00449	42	8	155	13,2	1,93
Gentil Rosso 4	TA00450	42	4	140	12,7	2,60
Gentil Rosso 48	TA00452	42	4	145	13,6	3,14
Gentil Rosso 58	TA00454	42	9	140	12,9	1,73
Italo Giglioli	TA00553	24	8	100	12,4	1,50
Mentana	TA00701	23	8	105	13,3	2,94
Mentana mutico	TA00705	33	8	125	12,4	1,88
Noè/A	TA00739	42	9	130	12,2	1,79
Noè/B	TA00740	33	8	135	12,9	2,09
O. 24	TA00755	33	0	115	12,4	3,64
Quaderna	TA00835	23	2	120	12,4	3,32
Quaderna	TA00836	42	7	145	13,1	2,48
Rosso Olona	TA00909	42	9	145	12,4	2,60
S. Michele	TA00945	40	0	130	12,4	4,25
Strampelli Francesco	TA01068	54	7	135	15,1	1,71
Tevere	TA01088	24	0	115	12,8	4,25
Tevere basso	TA01091	34	3	115	12,1	4,86
Villa Glori	TA01198	51	4	115	13,1	3,82
Villa Glori	TA01199	51	0	155	13,9	3,46
Vittorio Veneto/ 1	TA01217	43	2	145	12,9	4,23
Vittorio Veneto/ 2	TA01218	43	2	145	12,7	3,96

Supplementary Table S3. DON contamination levels (μg/kg) in 2023 and 2024.

		BG field 2023	VC field 2023	PV 1 field 2023	PV 2 field 2023	BG field 2024	VC field 2024	PV 1 field 2024	PV 2 field 2024
Ardito	TA00059	<10	<10	<10	<10	113.5	<10	<10	<10
Ardito mutico	TA00062	144.4	83.5	<10	<10	325.8	1034.2	<10	<10
Attilio Fabrini	TA00087	100.6	156.4	<10	<10	350.9	738.8	<10	<10
Brescia	TA00161	168.4	187.9	<10	15,1	740.0	1736.0	<10	<10
Cantore	TA00185	104.2	855.8	<10	<10	448.3	192.7	35.6	<10
Damiano Chiesa	TA00267	<10	141.1	<10	<10	609.9	406.7	<10	<10
Damiano Cremona	TA00271	12.6	202.5	<10	<10	153.5	402.9	<10	<10
Dante	TA00276	<10	68.0	<10	<10	145.6	148.1	100.7	<10
Edda	TA00958	185.4	310.3	<10	<10	268.4	251.0	62.8	<10
Edda	TA00959	108.1	18.1	<10	<10	270.1	262.9	<10	<10
Edda Sabina	TA00960	155.9	110.0	<10	<10	605.7	<10	<10	<10
Fausto Sestini	TA00354	265.2	213.7	<10	29,7	1893.4	345.0	128.6	<10
Fiume	TA00367	71.8	1139.5	<10	<10	421.9	523.7	<10	<10
Gentil Bianco	TA00440	478.6	267.4	<10	36.0	338.9	498.7	<10	<10
Gentil Rosso	TA00441	184.3	327.0	<10	<10	395.9	902.3	<10	<10
Gentil Rosso	TA00442	139.2	23.7	<10	<10	581.4	1643.2	25.6	<10
Gentil Rosso	TA00443	597.2	1296.3	<10	<10	1415.6	246.8	<10	<10
Gentil Rosso	TA00444	191.2	609.3	<10	<10	1123.1	239.3	119.2	<10
Gentil Rosso 13	TA00448	35.6	120.1	<10	<10	223.5	321.2	<10	<10
Gentil Rosso 160	TA00449	241.3	392.6	<10	<10	233.3	<10	<10	<10
Gentil Rosso 4	TA00450	<10	691.7	<10	<10	174.9	491.6	223.6	<10
Gentil Rosso 48	TA00452	268.5	<10	<10	<10	223.3	338.5	<10	<10
Gentil Rosso 58	TA00454	514.0	1450.8	<10	<10	851.5	589.5	<10	<10
Italo Giglioli	TA00553	36.2	157.2	<10	<10	362.4	187.3	<10	<10
Mentana	TA00701	32.0	189.4	93.1	<10	974.1	451.3	<10	<10
Mentana mutico	TA00705	61.4	135.8	<10	<10	498.1	1591.0	<10	<10
Noè/A	TA00739	90.0	611.4	<10	<10	834.6	423.6	19.2	<10
Noè/B	TA00740	97.3	427.0	<10	<10	410.2	97.4	<10	<10
O. 24	TA00755	45.0	225.8	<10	<10	183.3	129.7	<10	<10
Quaderna	TA00835	<10	<10	<10	<10	137.4	122.8	<10	<10
Quaderna	TA00836	56.6	279.3	<10	<10	243.2	914.7	58.3	<10
Rosso Olona	TA00909	156.7	232.6	<10	<10	288.2	435.1	373.2	<10
S. Michele	TA00945	287.1	287.0	<10	<10	352.8	<10	42.3	<10
Strampelli Francesco	TA01068	143.2	1561.8	<10	<10	181.4	352.2	<10	<10
Tevere	TA01088	47.0	<10	<10	<10	100.6	<10	<10	<10
Tevere basso	TA01091	<10	172.9	<10	<10	249.0	264.8	51.9	<10
Villa Glori	TA01198	22.6	50.5	<10	<10	154.1	<10	<10	<10
Villa Glori	TA01199	41.4	188.5	<10	<10	372.4	967.8	<10	<10
Vittorio Veneto/ 1	TA01217	74.5	517.3	<10	<10	207.1	557.5	<10	<10
Vittorio Veneto/ 2	TA01218	44.0	462.5	<10	<10	199.3	<10	<10	<10

Supplementary Table S4. TeA contamination levels (μg/kg) in 2023 and 2024.

		BG field 2023	VC field 2023	PV1 field 2023	PV2 field 2023	BG field 2024	VC field 2024	PV1 field 2024	PV2 field 2024
Ardito	TA00059	35.2	8.7	37.5	18.6	26.7	45.5	58.8	33.1
Ardito mutico	TA00062	312.2	660.8	18.4	11.6	45.3	111.7	665.1	8.2
Attilio Fabrini	TA00087	110.7	12.8	25.6	<5	29.9	27.6	30.9	146.8
Brescia	TA00161	27.2	48.7	87.3	36.8	33.4	35.5	51.0	59.3
Cantore	TA00185	180.4	61.0	25.3	27.0	19.9	21.0	17.0	37.2
Damiano Chiesa	TA00267	24.7	56.8	5.9	4.0	20.7	32.2	142.4	20.6
Damiano Cremona	TA00271	33.0	14.2	20.8	4.7	14.8	<5	19.3	40.8
Dante	TA00276	79.2	34.2	5.4	3.9	19.1	18.5	34.1	33.6
Edda	TA00958	146.9	184.0	9.4	17.5	65.5	37.4	34.0	43.6
Edda	TA00959	64.2	874.7	82.7	62.0	29.3	290.5	69.7	71.5
Edda Sabina	TA00960	105.9	291.4	4.6	63.1	37.2	49.9	100.0	55.0
Fausto Sestini	TA00354	132.1	199.1	9.0	61.4	108.6	106.4	121.0	195.9
Fiume	TA00367	160.9	33.8	19.8	3.4	26.1	12.8	93.7	40.1
Gentil Bianco	TA00440	203.7	55.1	<5	6.7	36.3	61.0	51.6	52.7
Gentil Rosso	TA00441	397.6	77.6	37.3	113.5	45.1	513.9	35.2	42.2
Gentil Rosso	TA00442	222.8	47.7	12.2	60.5	68.2	85.8	76.6	96.3
Gentil Rosso	TA00443	382.3	123.0	29.0	145.5	82.5	45.0	71.1	126.3
Gentil Rosso	TA00444	142.0	113.5	23.6	19.0	60.9	66.0	203.0	11.3
Gentil Rosso 13	TA00448	32.1	101.6	83.5	64.8	24.1	45.8	170.7	113.0
Gentil Rosso 160	TA00449	89.8	69.8	85.0	18.8	25.3	121.1	58.2	52.9
Gentil Rosso 4	TA00450	195.6	184.5	24.4	27.7	35.6	37.6	44.4	77.5
Gentil Rosso 48	TA00452	92.5	51.6	21.1	9.1	22.8	33.0	74.0	34.5
Gentil Rosso 58	TA00454	282.9	72.1	25.1	51.7	37.6	59.7	42.4	198.3
Italo Giglioli	TA00553	149.7	44.2	69.6	20.0	17.6	38.9	61.9	65.4
Mentana	TA00701	512.9	1681.3	33.1	48.7	410.8	611.5	487.3	436.7
Mentana mutico	TA00705	136.6	455.0	8.5	24.6	34.8	31.5	154.6	85.6
Noè/A	TA00739	261.4	40.4	65.6	22.1	52.7	40.5	56.3	45.1
Noè/B	TA00740	230.2	22.5	33.1	19.6	32.4	66.4	84.3	211.5
O. 24	TA00755	238.2	68.6	5.4	5.8	119.8	27.2	72.2	297.4
Quaderna	TA00835	29.1	43.8	49.6	49.7	52.8	41.9	33.3	46.5
Quaderna	TA00836	119.3	25.0	36.3	27.8	74.1	60.0	67.8	42.6
Rosso Olona	TA00909	101.0	114.1	43.4	15.4	63.5	28.6	54.1	48.2
S. Michele	TA00945	172.6	66.0	6.3	13.7	113.6	136.3	84.0	133.6
Strampelli Francesco	TA01068	466.5	96.9	152.8	32.2	132.6	55.9	66.6	91.4
Tevere	TA01088	174.4	56.0	81.4	4.4	200.6	36.2	108.4	55.1
Tevere basso	TA01091	90.3	86.4	26.9	6.3	68.6	87.9	136.6	42.1
Villa Glori	TA01198	24.9	94.3	29.7	<5	20.5	85.3	28.2	14.2
Villa Glori	TA01199	122.4	60.5	26.4	21.6	37.4	48.0	22.9	55.8
Vittorio Veneto/ 1	TA01217	128.9	69.0	7.8	18.1	43.8	56.5	36.5	69.7
Vittorio Veneto/ 2	TA01218	116.5	155.3	10.3	15.7	32.8	24.7	141.3	73.1

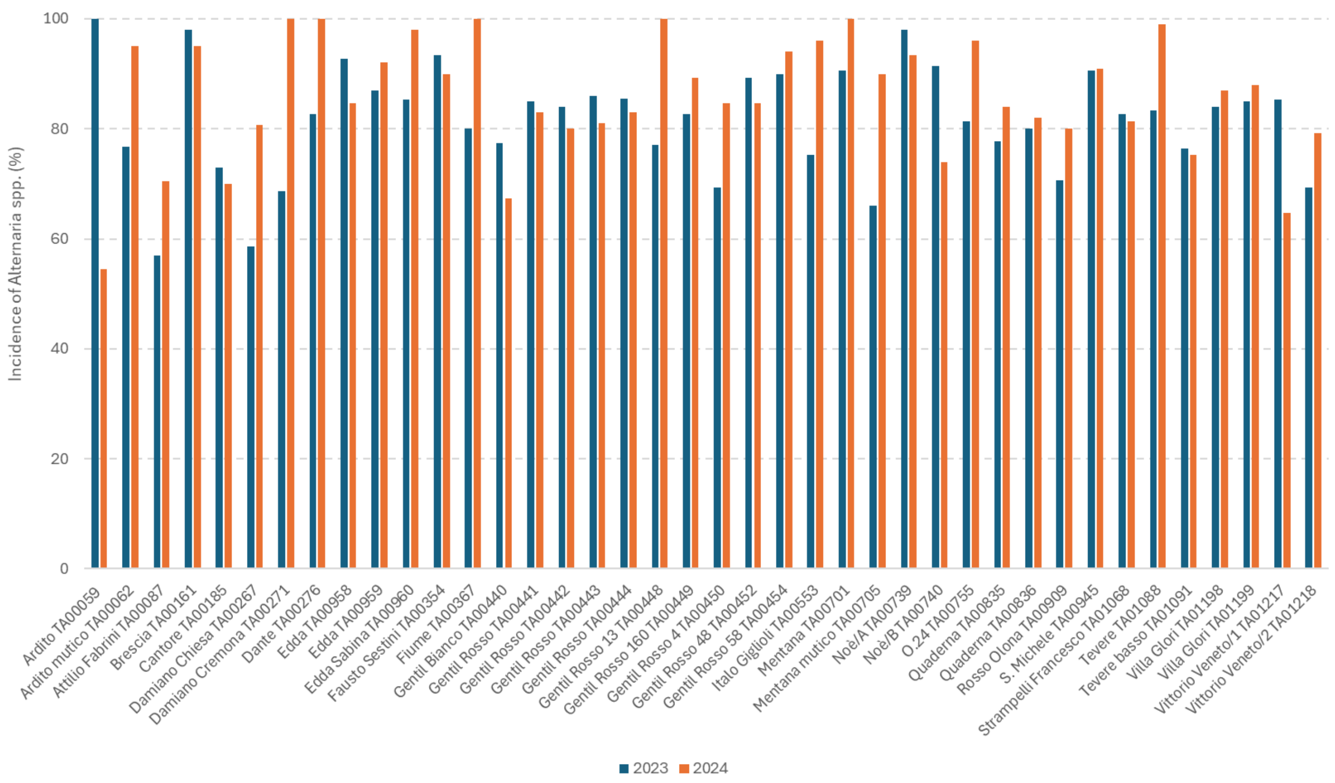

Supplementary Figure S1: Total fungal incidence in the 40 heritage wheat varieties analyzed in this study during years 2023 and 2024. For each variety, the values represent the mean across four locations. Incidence was calculated as the total number of fungal isolates by the total number of kernels analyzed.
